# Value-based pricing for advanced therapy medicinal products: emerging affordability solutions

**DOI:** 10.1007/s10198-021-01276-2

**Published:** 2021-06-09

**Authors:** Elisabete Gonçalves

**Affiliations:** CTI Clinical Trial and Consulting Services, Lisboa, Portugal

**Keywords:** Advanced therapy medicinal products, Health technology Assessment, Value, Pricing, Affordability, I18, H42, H75

## Abstract

The emergence of advanced therapy medicinal products (ATMPs), a disruptive class of health technologies, is generating important challenges in terms of value assessment and their high prices introduce critical access and affordability concerns. The aim of this article is to analyze the challenges of traditional value assessment and price and reimbursement methods in the evaluation of ATMPs and to characterize the current and prospective financing solutions that may ensure patient access and affordability for these health technologies. Standard Health Technology Assessment (HTA) is not designed for ATMPs, and may delay access to these health technologies, thus a broader concept of value is required. As a consequence, value-based pricing methodologies have been gaining terrain to cope with the specific challenges of ATMPs. The pricing and reimbursement framework should ensure the balance between encouragements to innovation and maximization of value for money for payers, through the attribution of a fair price to new health technologies. Early scientific advice by regulatory and HTA bodies to developers is key, as it will contribute to diminish the perspective gap between developers, regulators and payers. The high efficacy/high price dynamic of many advanced therapies will demand novel financing models, both in the EU and US. Managed entry agreements (MEA), with financing being conditional to the submission of additional evidence, associated with methods of leased payments, may offer effective strategies to address the uncertainties caused by the evidence gap associated with ATMPs, ensuring affordable and sustained access.

## Policy framework

Advanced therapy medicinal products (ATMPs) are a category of highly innovative and complex biological products and the classification of these products differs between regions. In the EU, there are four major classes, gene therapy, somatic cell therapy, tissue-engineered therapies, and combined advanced therapies, while in the US two groups of products are defined, gene therapy and cellular therapy [1]. The factors that make ATMPs unique include their potentially curative nature, aligned with lifetime benefits, potential long-term safety issues, organizational and scaling issues, and a significant up-front cost for payers [2].

In the EU and US, the regulation of ATMPs falls under the biologic products licensing scope of the European Medicines Agency (EMA) and of the Food and Drug Administration (FDA), respectively. Both agencies have established a regulatory framework that promotes the development and approval of these products [3].

Additionally, the EU Regulation on ATMPs authorizes the use of non-authorized ATMPs under the certain circumstances. This hospital exemption rule requires application in each EU Member State individually [4].

The hospital exemption scheme should only be applied in exceptional conditions, where no equivalent authorized ATMP is available on the market and the product is adequate for use in the individual patient. An inappropriate use of the hospital exemption may discourage the submission of marketing authorization applications for ATMPs [5].

The dissemination of novel technologies usually follows an S curve market penetration pattern and ultimately reaches saturation level. Considering the promising number of early clinical successes, it can be considered that ATMPs development recently progressed through the early adoption stage.

However, due to commercial reasons, several approved ATMPs have seen their marketing authorizations withdrawn by their developers [6].

### Value assessment

From an economic standpoint, the value of a good or service to an individual is what that individual would be willing to pay for it in monetary terms, or give up in terms of other resources or time, to receive it. The value also therefore represents the ‘opportunity cost’, given that the individual has sacrificed the opportunity to use those resources in their next best alternative use [7].

In terms of ATMPs pricing, it is not just a question of defining how much payers are willing to pay for these products, but defining the value and economic justification for a given medicinal product.

Innovative medicinal products, medical devices, and diagnostic tests differ from the inputs of hospital and health care professionals, since the knowledge they represent may be used worldwide, being considered a ‘global public good’. From this it can be inferred that every individual in the world could be willing to support, to some degree, the underlying research that creates them [7].

The willingness to pay demonstrated by payers is often determined through Health Technology Assessment (HTA). However, for many ATMPs, it is not viable to calculate the incremental cost-effectiveness ratio (ICER) [8].

Several dimensions have traditionally been taken into account when assessing the value of new pharmaceutical products, namely, the improvement in length and quality of life, ease of use, and cost-savings to the health system [9].

It has been defended by a Special Task Force of the International Society for Pharmacoeconomics and Outcomes Research (ISPOR) and several authors that potential novel elements of value should be considered in the assessment of ATMPs, such as the value of knowing, fear of contagion, insurance value, value of hope, real option value, equity and scientific spillovers, and also health equity, lifetime burden of illness, caregiver burden, family spillovers and socioeconomic impact [10, 11].

In the scope of rare diseases, that many advanced therapies target, core elements of value, relevant to pricing and reimbursement decisions, have also been identified. Firstly, at the patient level, survival, morbidity, treatment options, side effects and treatment convenience. Secondly, at the healthcare system level, health care budget and organization and finally at the societal level, family/carer health-related quality of life, family/carer economic burden and societal economic burden [12].

The majority of countries adopt a ‘health system’ perspective, instead of a wider societal perspective when making pricing decisions, therefore they mainly consider costs for healthcare payers and added therapeutic value for patients in HTA [9].

However, it has been argued that the HTA analysis should follow the societal perspective, as indeed these technologies, by allowing the possibility of cure, often in pediatric patients, enable great benefits for society and, therefore, broader elements of value should be considered in the analysis. Although not yet possible to quantify, these additional elements of value could be considered in the HTA analysis and subsequent price negotiation [13].

There are several factors that influence policy decisions on reimbursement in Europe, including the disease burden, the size of the target population, and the level of unmet need. As the level of unmet need increases and the size of the target patient population decreases, the willingness to pay will increase [14].

Value-based assessments allow to link therapeutic benefits for the patient and the healthcare system and the willingness to pay and adopt the new technology. The most common method by which added value is captured and translated into a reimbursed price employed across all the biggest EU markets is the magnitude of the incremental clinical benefit. Economic factors are then considered, such as cost effectiveness and budget impact [15].

There are considerable challenges associated with assessing the value of ATMPs for pricing and reimbursement. Many ATMPs currently in development target rare diseases/indications with small patient populations, which greatly increases the difficulty in generating sufficient clinical evidence to support significant health improvement claims. Also, the long-term effectiveness of potential curative therapies may be unknown, and decision makers may lack the sufficient information to adequately evaluate ATMPs using current pricing and reimbursement methods, consequently risking the under or over-valuation of these products.

Since ATMPs have the potential to cure or provide substantial health benefits to patients with seriously debilitating or life-threatening diseases, it may be considered unethical to deny patients a potential cure, in the period the evidence development programs are being finalized [16].

The majority of advanced therapy approvals in the EU and US have occurred over the previous 7 years, target rare indications with few or no existing treatment alternatives, and few relevant products exist to be compared to the new products in this class, which adds complexity to HTA deliberations [13].

Society may attribute more value to curative therapies than treatments that offer the same ‘total’ health gains through marginal gains over many years and/or patients, since curative therapies possess the potential to eliminate the need for long-term chronic treatments and provide longer term increases in quality of life. There is currently, however, scarce evidence available that suggests that this preference does exist, and this premise is not currently included in HTA [11, 17].

For ATMPs, a crucial question to be asked is: “how does one value a cure?”.

There have been numerous attempts to define the term “cure,” with conflicting views on what constitutes a cure and on how much time must pass before declaring a treatment is a cure [18].

Given the intrinsic characteristic of many ATMPs of offering a potential cure, it is important to define what is meant by “healthcare cost cure”.

The concept differs from three definitions of cures found in the literature: absolute cure, functional cure, and statistical cure. An absolute cure is manifested by complete and final termination of a specific disease. In a statistical cure the mortality of the patient returns to the rate present in the general population. Lastly, a functional cure is considered when the disease is controlled and its manifestations are no longer present. A healthcare cost cure represents a higher standard, since it considers both the cost and health effects of the intervention, namely the long-term medical follow-up in the cases of functional cure. Payers express uncertainty about long-term outcomes of cures achieved with ATMPs and many years of follow-up of the patients might be involved [19].

So far, most ATMPs have been approved with a narrow license indication and frequently for use in second lines of treatment. In this scenario, there is increased reliance on indirect comparisons using historical controls, lack of long-term outcome data, including health-related quality of life and survival, and dependence on endpoints that may not be good surrogates for outcomes significant to patients, which may add significant bias [8].

Indeed, the use of real-world evidence (RWE) for value assessment is not devoid of challenges, since this data is not usually collected to assess key dimensions of value, data consistency and completeness may be an issue [20].

In this context, outcomes modeling provides a way to bridge the gap caused by the absence of suitable data from clinical studies of short duration or RWE. Hence, for many ATMPs, extrapolations and indirect comparisons prove to be especially relevant. Indirect comparison is most critical for ATMPs in cases where the comparator in the pivotal trial is not aligned with the standard of care in the country under assessment, or in cases where ethical concerns call for the performance of a single-arm study. This methodology enables the consideration of data from observational sources, such as registries and meta analyses, to assess the comparative effectiveness of the new health technology [15].

It is broadly recognized that the clinical benefit of ATMPs can extend over a longer horizon than is obtained by clinical trial data, and considering this extended value is crucial to capturing the full worth of these therapies. This task usually comprises multiple parametric and non-parametric models that are validated through statistical models and clinical expert opinion to assess biological plausibility [13].

Since they are able to provide sufficient flexibility to incorporate all value elements, multi-criteria decision analytic frameworks have been proposed for rare diseases, that many ATMPs target [12].

Some HTA bodies show more willingness than others to admit new types of evidence beyond randomized controlled trials, or to consider economic models that include extrapolating longer-term benefit from limited existing data.

In 2019, ICER has revised its Value Assessment Methods for High-Impact “Single and Short-Term Therapies” and decided to consider in the analysis additional elements of value, although it proposes no quantitative integration of these elements into the value assessment framework of advanced therapies. The first element captures the “value of hope,” namely the value of having the choice among treatments with a different balance and timing of risks and benefits [18].

A new potential benefit or disadvantage related to the option of receiving future treatments is also proposed. The potential advantage is linked to option value, that is the ability to benefit from future treatments that the patient might not otherwise have been able to receive. The possible disadvantage is that some ATMPs might trigger immune responses, that could deem impossible the treatment with a future generation of products [18].

Although the evidence available corroborates the importance of integrating the views of the patients in HTA, at present, there is no consensus on what “patient-centric HTA” actually implies. In the context of a private insurance-based health system, in which an important proportion of the therapeutic payments may be performed directly by the patients, the consideration of the patient perspective in HTA might be more relevant, than in a publicly funded national health system [21].

Patient-reported outcome measures (PROMs) have been considered a helpful source of data that should serve to inform HTA. Similarly, patient-reported experience measures (PREMs) that aim to capture elements that include burden of disease, route of administration and impact on caregivers, might be considered. In the US, FDA has endorsed the use of PROMs to support label claims for regulatory decisions, yet there is still limited evidence of their use by HTA bodies [21].

### Value-based pricing

The difference in the definitions of cost, price and value is important to highlight. In the context of pricing and reimbursement, cost is the amount required to manufacture and deliver the technology, price is the amount reimbursed, and value is what the healthcare provider perceives as the worth of the technology. These elements are, to some extent, positively correlated, but price and cost should be mostly separated.

The price of a health technology should depend largely on external forces, such as market value, orphan status, clinical value in relation to competitors, and other health economics parameters. On the other hand, although cost may be a factor in pricing evaluation, it should not be a primary driver [13].

Price is, without a doubt, a critical feature of ATMPs [8] and in terms of prices worldwide, a large variability was found, with the highest prices of advanced therapies being found in the US [22].

Groundbreaking technologies, such as ATMPs, can potentially benefit all inhabitants of Earth, and consequently, have the characteristics of a global public good. It has been defended by economists that for this type of economic goods, the optimal research and development financing system should be based on global differential pricing across countries, based on the different ability and willingness to pay for health improvements. There is a precedent for this situation with treatments for HIV and vaccines, in which cases, global access and differential pricing across jurisdictions were implemented [10].

Also, in the cases a new technology is able to offer the cure of a disease that would otherwise prove to be fatal in early childhood, a question emerges about the value, that will translate into the cost, of a full life [10].

A fair pricing will be a pricing that ensures a proper and socially acceptable reward for developers of disruptive innovation, while ensuring affordable access to the best possible treatments for all patients. It is accepted that, due to the distinct characteristics of ATMPs and the diseases they address, no single, one-size-fits-all solution will be applicable in the price definition [23].

Since ATMPs are likely to reach the market with immature evidence of their effectiveness, HTA bodies and payers are increasing the scrutiny on the actual incremental value of innovative therapies claiming high prices [24]. The concept of ‘de-linkage’, by which the price of a new health technology is disconnected from its claimed research and development costs has been discussed as an important reform on how innovation should be funded [9].

Parallel to the recent shift in the US policy, the assessment of reimbursed pricing for ATMPs in the EU has shifted in the direction of value-based models [14].

Ultimately, drug pricing is negotiated at a national level and healthcare authorities have divergent decision-making frameworks in place [13]. In the largest European countries (Germany, United Kingdom, France, Italy and Spain), ATMPs follow the same pricing and reimbursement pathway than conventional medicinal products [15], however there is diversity in how HTA is performed, with the additional layer of complexity that is brought by regional-level pricing decision-making [13].

Factors, such as the contribution to Gross Domestic Product (GDP), involvement of patient advocacy groups as well as ethical, equality, and equity considerations also impact on the final decision on a country level. Benchmarking should be performed for price determination, against existing alternative care, if no direct comparator is available.

However, it has been proposed that ATMPs with a hospital exemption should be excluded from the benchmarks for price determination [25].

Value-based pricing is broadly considered the method of choice for determining the price of new health technologies [26]. This method is linked to a welfarist framework in which consumers opt from different private health plans that offer distinct coverage/premium choices, or taxpayers select an annual health budget following a political procedure [27]. It is considered to present advantages when compared to most of the alternatives, namely price negotiations, or internal or external reference pricing. Following this approach, the price of the new product is determined by the maximum price that would result in the ICER of the new alternative, when compared to current clinical practice [26]. If the determined price is cost effective, under a specified threshold (cost per quality-adjusted life-year [QALY]) the health technology should be reimbursed. Ideally, value-based pricing can be built to attain the highest health gain for a given budget and establish heightened incentives for investment on research and development [27].

It has been argued that in some cases, where the new technology offers a cure, the calculation of value-based pricing might result in a price value too high, which can raise serious affordability issues or too low, when it is recognized that rewards should be allowed to manufacturers who develop an alternative for highly expensive therapies that greatly impact life expectancy or quality of life [26].

At present, the market for ATMPs is limited and direct competition to currently authorized products may not exist. The health technologies that first reach the market possess a clear competitive advantage in their pricing strategies and increase the barriers to success for subsequent competitors, which to replace an existing treatment must demonstrate superiority [13].

Another relevant aspect in ATMPs pricing is the discount rate used by HTA. The discount rating takes into consideration the relatively higher value of clinical outcomes in the present when compared to those in the future. Curative treatments, such as advanced therapies, are highly sensitive to the discount rate used, due to the prolonged nature of their clinical benefit [13].

Along with effectiveness, safety concerns may arise for ATMPs and given the novelty of these technologies, the uncertainties in terms of long-term safety should be considered in the negotiated price.

Some advanced therapies, like gene therapies, may originate significant cost offsets as they replace expensive existing long-term treatments, such as enzyme replacement therapy, reducing hospitalizations and preventing further adverse clinical events, potentially expensive. It is crucial that these long-term savings are included in affordability considerations, to measure the full financial impact of introducing ATMPs [17].

Importantly, the total costs of the treatment with the ATMP is likely to far exceed the product theoretical acquisition cost [8].

Given the nature and uncertainty associated with ATMPs, value assessment and pricing and reimbursement decisions should allow changes in terms of pricing, both upwards and downwards, as new evidence on value becomes available for these products [12].

Traditional pricing mechanisms for health technologies include External Reference Pricing and Internal Reference Pricing. External Reference Pricing, where a change in the price for a product in one country will affect the price in other countries, is widely used amongst European countries.

This pricing mechanism offers developers an incentive to delay the launch, or not launch at all, in lower-priced countries to avoid the expected negative impact on prices in other countries [9].

Another challenge is the fact that the costs of ATMPs gathered from specific hospitals in a given country might limit generalizability to other jurisdictions, due to different methods of production, pricing and service delivery in different geographies [2].

Internal Reference Pricing, on the other hand, performs a benchmarking of prices of existing products in the same therapeutic area or with a similar mechanism of action in the same country [3].

It has long been discussed that the lack of transparency and information asymmetry in the pricing mechanism in the different countries creates several difficulties, namely when external reference pricing is used. The ‘real’ prices are often unknown, as confidential discounts are frequently agreed with the payers [9].

However, some have argued that transparency on costs and price-setting should be incentivized, rather than enforced, as it should not be imposed as a stand-alone measure.

Indeed, the obligation to communicate all prices and costs could have the opposite effect on access and affordability, particularly in middle and lower income countries [23].

However, it is important to note that pricing is not the only barrier to market ATMPs face. Other aspects, such as complexity of use and disruption to standard patient journey, may affect the uptake of these products [13].

To ensure a viable pricing decision, sponsors should, from an early stage in the product development, conduct analysis to assess the willingness to pay, by consulting payers, health care professionals and patients.

As the technology of ATMPs matures, it is expected that, over time, costs may decrease in a significant manner, as it was the case with monoclonal antibodies, driven by increased manufacturing capacity, greater efficiency in manufacturing capabilities, and further implementation of efficacy-based pricing models [28].

### Access and affordability solutions

Advanced therapies embody a significant change in the healthcare reimbursement paradigm, since most current treatments for serious conditions involve chronic palliative care that allows incremental improvements and/or temporary suspensions in the disease progression [13].

Payers are facing the critical challenge of ensuring a balance between the financial sustainability of the healthcare systems while encouraging the innovation and development of new therapies, that address unmet needs and catastrophic diseases [29].

Currently, payers largely use a combination of cost effectiveness and budget impact to tackle with affordability issues, mainly applying a ceiling [30].

However, with the traditional payment model, there is the risk that the goal of financial solidity of the health systems can only be achieved at the expense of patient access and incentives for innovation [18].

To ensure patient access to these novel therapies, the development and implementation of adapted payment models, that enable payers to support the costs of these therapies, is thus, paramount [13].

A report from the European Commission on «Innovative payment models for high-cost innovative medicines» has proposed basic principles for new payment models. These principles include, among others: greater price and cost transparency, development of methodologies to measure the value of pharmaceutical products, setting of better rewards for higher therapeutic added value, exploring non-linear payment systems and stimulating the creation of dialog platforms [31].

In the context of value-based pricing, a competition arises between what agreement is cost-effective and what the payers are able to afford. This dilemma has been referred to as the affordability barrier, and it occurs in both insurance-based health care systems and politically controlled health care systems [18].

A new health technology is “affordable” in the scope of the existing budget if it creates value exclusively by reducing existing medical costs, with no increase in future costs or current or future QALYs. If, in opposition, a new therapeutic option value originates primarily from reducing future medical costs and/or allowing QALYs gains, current or future, affordability issues arise. The problem is exacerbated as the potential treatment population increases [27].

To fulfill the financing needs of health technologies with the characteristics of ATMPs, for which health benefits are expected over a longtime horizon, a combination of outcome-based agreements and potential methods of leased payments can be applied.

It is relevant to note that the spread of payments over an agreed period of time are common for medical equipment, where the cost is spread over the time horizon in which the equipment will be in use [11].

Although payment based on outcomes would allow payers to share the risk of uncertainty with producers, several legal and logistical hurdles need to be faced, to implement such agreements. Very importantly, the clinical outcomes that constitute a cure will need to be defined, agreed and measured in an objective manner, and the length of time required for the patient to be considered cured must be defined. In practice, patients will need to be properly monitored to guarantee the clinical outcomes set are, indeed, met. If not correctly defined and implemented, this monitoring procedure may require a considerable amount of resources and create an administrative burden on healthcare systems.

Managed Entry Agreements (MEA) stipulate, frequently in a confidential manner, the conditions under which a health technology will be priced and reimbursed, in a defined population of patients [32].

They are associated with considerable administrative costs, and their application should be accompanied by a disinvestment strategy, in the scope of price or reimbursement revisions [9].

The MAE that are commonly considered for ATMPs are population (indication) specific arrangements, agreements based on financial risk, and outcomes-based agreements. Population (indication) specific arrangements limit the financing to a subpopulation of patients, e.g., per indication, or by a defined severity level. The objective will be to ensure cost-effective use of the treatment by confining the access to a subpopulation that, theoretically, can attain the highest value for the treatment. On the other hand, agreements based on financial risk aim to make the cost to the payer foreseeable and controllable within the budget framework. These schemes are not linked to health outcomes and may include patient spending caps, stopping rules, among others [16].

Outcomes-based agreements are divided into two groups: conditional coverage/reimbursement and performance-linked reimbursement schemes. In conditional coverage schemes the coverage is granted conditionally upon the generation of RWE from clinical practice. Once additional evidence is gathered, prices and reimbursement may be re-negotiated. Conditional coverage schemes are divided into two categories, coverage with evidence development at the population, or at the individual level. In performance-linked payment or reimbursement schemes, on the other hand, the financing is linked to a measure of clinical outcomes [16].

Since long-term effects of advanced therapies are uncertain, there is a strong argument for establishing future payments that are dependent on the actual health outcomes and savings achieved. This perspective will shift outcome risk from the payer to the producer, and will align the incentives of the producer to design a health technology with an optimized long-term benefit-risk profile [27]. In line with this, amortization or leasing schemes have been proposed for advanced therapies, where up-front payment systems are substituted with several payments divided over the expected duration of benefit provided by the health technology [11].

Spreading the payment over multiple years presents several advantages in terms of coping with affordability issues. On one hand, this allows to opt for treating more severe patients first, which are the ones at higher risk of substantial short-term medical expenses. On the other hand, the delay in the treatment of some patients may provide the time for competing health technologies to be launched, allowing payers an opportunity to negotiate discounts on prices. Lastly, the first health technology to be launched in a new class is not always the most effective and, therefore, patients who delay treatment could benefit from a wider range of treatment options [27].

Contracts with payments delayed in time promote the alignment of the payment and benefits over time and shifts performance risk to the producer, who is better positioned to control the performance of the health technology. It is worth emphasizing that these agreements have the potential to distort incentives for payers, if the current payer is allowed to shift payment disproportionately toward future payers, who were not involved in the contract definition [27].

It is not completely clear whether amortization will emerge as a feasible option for managing the affordability of advanced therapies. Concerns have been raised that the introduction of such agreements might promote higher prices and thus compromise the sustainability of the health care systems. Nevertheless, there are characteristics that make some advanced therapies more suited than others for an amortization approach. These characteristics include: a one-time or very short-term treatment with a curative impact, a robust clinical benefit that can be monitored through an outcomes-based process, a population size large enough to raise apprehensions regarding up-front payments, and finally a method by which the regimen price is fairly defined to reflect the added value brought to patients [17].

In the biggest European countries, private insurance does not offer a significant advantage over public reimbursement in terms of access to ATMPs. Unlike the US, in these countries, healthcare spending is mainly motivated by public health insurance rather than private payers [14].

To ensure the continued access to patients of new technologies, producers, HTA bodies and payers should cooperate at a national level to optimize forecasting and collaborate at the European level for horizon scanning with the purpose of helping national payers forecast and plan for expenditure and ensure adequate funding of innovative technologies [12].

Italy already uses risk-sharing reimbursement approaches relatively frequently, where discounts and rebates are delivered in response to certain clinical milestones [13].

In Germany, outcome-based payment agreements have already been implemented for CAR-T cell therapy. In this country, annuity payments have been recognized as a possible answer to the problem of high up-front costs and undefined long-term clinical outcomes. The possibility of German patients to switch insurance creates some apprehensions among sick funds regarding the affordability of ATMPs in the upcoming years. To alleviate the risk of patient mobility, a basis for risk-sharing could be proposed and utilized among a consortium of insurers, as it has been performed for CAR-T cell therapy. Risk-sharing in the manner of collectivization, like as the use of 'high-risk funds', can allows initial costs to be collectivized and insured, allowing patient access and safeguarding payer affordability [33].

The US face unique challenges in the pricing and reimbursement of cell and gene therapies.

Indeed, unlike most EU countries, in the US private health insurers assure reimbursement to the healthcare costs of individuals. It is frequent that the insurance provider of an employer, and consequently of its employees, are altered roughly every 2–3 years. The development of annuity reimbursement models is more challenging in this dynamic scenario, since annuity payment contracts will need to be transferred between insurers, that will require a connected provision of service [13].

In the US, the amortization options are divided into categories depending on who is responsible for the financial risk and who offers the funding. The major amortization categories are: consumer loans, third-party financial institution financing for payers, government financing for payers and manufacturer-managed financing, in which long-term payment plans are agreed with payers and most likely linked to outcomes-based agreements [17].

Globally, it is important to clarify the relationship between value-based pricing and outcomes-based contracting. Indeed, an outcomes-based contract per se is not related to value, if the price is not set through a value-based methodology.

## Conclusions

The intricacies of the clinical effectiveness and safety, regulatory framework, as well as the economic and ethical issues brought by the groundbreaking innovativeness of ATMPs, still require further study and discussion, while manufacturers, regulators and payers are pressured by patients, who demand a timely access to the most effective and innovative treatments.

The problematic of the high prices of ATMPs has distinct contributing factors and hence should be approached through a combination of regulatory and policy measures and cooperation between all stakeholders. Ultimately, a global perspective on the development and reimbursement of these health technologies is needed, given their unique positioning as economic goods.

Overall, pricing and reimbursement decisions should balance the right to receive the return of investment by developers, promoting continued research in novel health technologies, with the necessity to maximize value for money for payers.

Pricing and reimbursement decisions should be value-based, yet they should also present flexibility, since the value of a health technology will suffer alterations with time and vary according to the geographies.

Manufacturers should engage with the different HTA stakeholders at an early stage of the product development to ensure the clinical development strategy aligns with key parameters in pricing and reimbursement appraisals in the different countries. Consequently, integrated evidence planning, has become essential to align the evidence generation activities across product development, regulatory submission, RWE, HTA and the entire product life cycle.

An increase in value-based payment models and innovative agreements is expected, as developers, payers, and policymakers align on strategies to address the current effectiveness data gap and the required regulatory framework optimization. Indeed, managed entry agreements, that are able to reward effective treatment, embody a reasonable direction in assuring patient access in the near future, with financing being conditional to the submission of additional evidence, and payments being aligned with genuine savings to the health care systems. Still, the feasibility of collecting additional safety and effectiveness data on the long-term effects of these health technologies may greatly influence the successful implementation of these agreements.

However, simple managed entry agreements might not provide an adequate answer for affordability challenges, and innovative financing mechanisms will be needed to guarantee affordability. Indeed, financial agreements that include annual payments over a defined period of time, that are affected by the successful outcomes of the treatment, are likely to be present in the access decisions for ATMPs in the coming years.

Globally, to ensure timely and continued patient access to new groundbreaking technologies, while safeguarding affordability concerns, health technology policy should be approached as a broader concept that goes beyond pricing and reimbursement frameworks. In addition to HTA, other supporting frameworks such as pre-launch activities, as horizon scanning, and post-launch procedures, will help to support the access policies implemented.

It is not expected that one single financing approach will be appropriate to all new ATMPs, and the continuous flexibility and cooperation of all stakeholders will be crucial to ensure successful and continued access.

The growth in the access to advanced therapies will likely require difficult ethical decisions regarding which treatments should be reimbursed, especially when these health technologies are used for diseases with a larger patient population.

On the other hand, if the investment and dedication to develop cures for life-threatening diseases are not properly rewarded, there is the risk that developers may opt to concentrate their efforts on incremental instead of breakthrough treatments, in detriment of public health benefit. However, it is also anticipated that the prices of these health technologies will decrease, once development and manufacturing technologies reach a higher maturity level.

In conclusion, the financing of advanced therapies will likely require creative thinking, especially on the part of policy makers, and the current business model will need to be revised to adapt to the challenges created by new paradigm-changing therapies. Indeed, innovation will need to arise from the payers, as much as it originates from the developers of novel therapies.
